# Adrenalin and Cocain

**Published:** 1903-05-15

**Authors:** Marcel Lermoyez


### ADRENALIN AND COCAIN.

BY MARCEL LERMOYEZ, M.D.

It should not surprise us in these days to be able to perform neatly, without hemorrhage, those nasal operations which up to the present have always caused such bleedings as to merit the epithet of "gory." Already on several occasions, with only the assistance of cocain and adrenalin, I have been able to correct deviated septa, without more effusion of blood than if I had made simple examinations; I have been able to scrape right down to the base of an ulcer of the nasal mucous membrane; only yesterday I executed, without any other serviette than the patient's handkerchief, a removal of the inferior turbinated bone, an excellent operation. which, however, one often hesitates to perform for fear of certain copious hemorrhage. At the present moment, all the rinologists who have tried adrenalin are astonished and thoroughly satisfied.

I will refer only to its prodigious blanching property in comparison with all known hemostatics, which arrest the flow of blood; adrenalin alone is able to prevent it.

Cocain and adrenalin are two substances of similar destiny, and surgery will treat them as twin remedies; one pleases the patient in destroying pain, the other the operator in suppressing hemorrhage. They assist each other, for cocain, which has not much action alone on inflamed tissues, easily anesthetizes them once adrenalin has blanched them to the normal condition. Their simultaneous employment may thus be summarized: no pain, no blood. Elsewhere, and frequently, in the text of this journal, adrenalin will be the subject of scientific studies, more carefully carried out. To-day I have only wished to call your attention for a moment to a new marvel, and I can not better present adrenalin to the surgical world than by saying that its preventive hemostatic power has earned for it the name " The alkaloid of Esmarch's bandage."—*Therapeutic Notes.*

